# Protraction of Mandibular Second Molar for Substitution of Adjacent Missing First Molar With a Mini-Implant-Anchored Albert Loop Appliance

**DOI:** 10.7759/cureus.58397

**Published:** 2024-04-16

**Authors:** Hong Zhou, Xuechun Yuan, Huiyi Hong, Wenli Lai, Hu Long

**Affiliations:** 1 Department of Orthodontics, West China Hospital of Stomatology, Sichuan University, Chengdu, CHN

**Keywords:** accelerated orthodontic treatment, dental appliances, mandibular molar protraction, orthodontic mini-implant, biomechanics

## Abstract

Protraction of mandibular posterior teeth into edentulous regions is challenging in clinical practice. This case demonstrated a minor tooth movement of a mandibular second molar to substitute its adjacent missing first molar in a 15-year-old female. An efficient bodily movement of the mandibular second molar was achieved through a mini-implant-anchored protraction loop appliance. With this carefully designed biomechanical system, over 10-mm molar protraction was accomplished within 14 months without mesial or lingual tipping. The adjacent third molar erupted spontaneously during the protraction process and drafted mesially. Through brackets and segmented archwire after the protraction, the second and third molars were successfully protracted and good buccal interdigitation was achieved. The combination of the Albert protraction loop and mini-implant allows for more efficient protraction of the mandibular molars, avoiding mesial tipping and lingual rotation of the molars.

## Introduction

Caries, periodontitis, and failure of teeth eruption often lead to loss of teeth, in which loss of mandibular molars is frequently encountered in clinical practice, and it may lead to undesirable consequences, such as tipping of neighboring teeth, supra-eruption of opposing teeth, occlusal trauma, temporomandibular joint dysfunction, alveolar bone loss, etc. [1,2]. In addition to prosthesis restoration such as implant, orthodontic protraction of second and third molars to substitute the missing molars is a viable method [3].

To date, there are several treatment modalities for molar protraction with conventional biomechanics, such as U-curves and upright springs [3-5]. However, these methods may lead to anchorage loss of anterior teeth, resulting in reciprocal movement of anterior teeth. Meanwhile, a roundtrip and slow protraction speed may result in a long treatment duration [6]. A relatively new approach is to use mini-implants to reinforce anterior anchorage and expedite tooth movement [3].

Successful molar protraction with mini-implants was reported in several case reports [7,8]. However, mini-implants are unable to eliminate all potential adverse effects like mesial rotation and lingual tipping of the posterior teeth [7,9].

In our case, we will present a successful molar protraction case with a carefully designed force-and-moment system built on a mini-implant. The Albert loop appliance efficiently protracts the molar while also preventing mesial and lingual tipping of the tooth. The study will also discuss the biomechanics of mandibular molar protraction.

## Case presentation

Diagnosis and etiology

A 15-year-old female patient presented to the hospital with a chief complaint of a missing tooth. She had a mild convex facial profile with a small nasolabial angle. Intraoral examination revealed that #46 remained only two residual roots, which left approximately a 10-mm space between #47 and #45.

**Figure 1:**
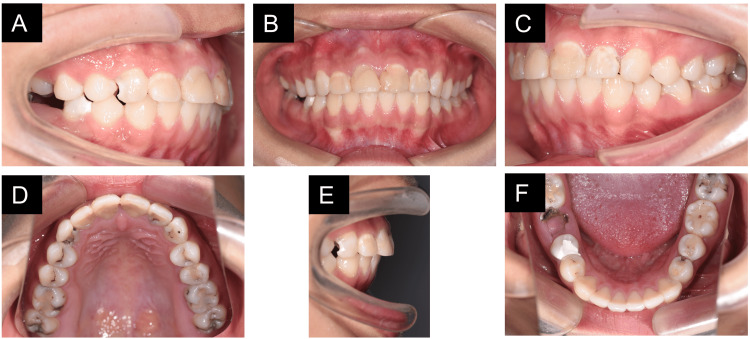
Pretreatment intraoral photographs. (A) Occlusion of the right side, (B) anterior photo, (C) occlusion of the right side, (D) upper dentition, (E) overbite and overjet, and (F) lower dentition.

**Figure 2:**
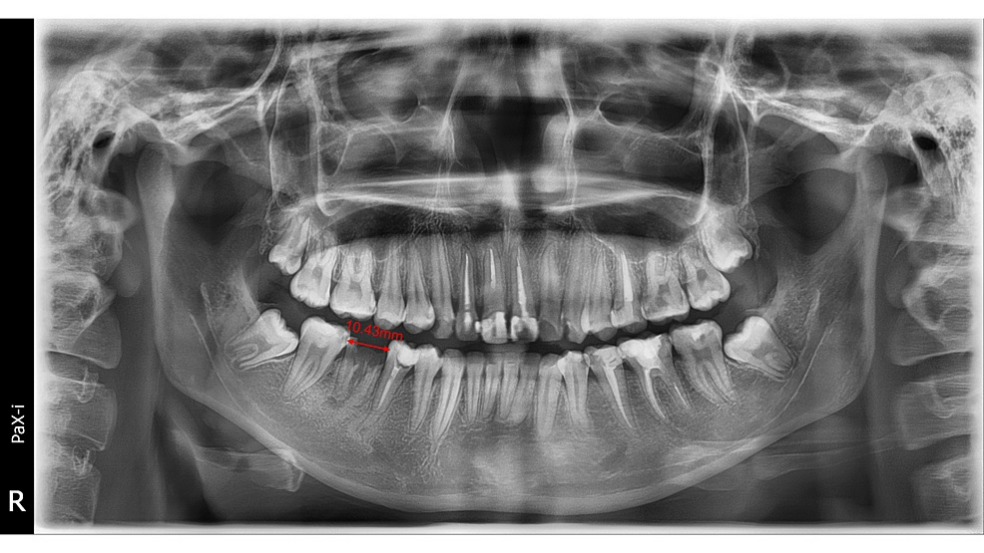
Pretreatment panoramic radiograph. It showed unrestorable #46 and the space between #47 and #45 was 10.43 mm.

Normal overjet and overbite were observed, along with a bilateral Class I canine relationship and a Class I molar relationship on the left side. There was no crowding, and the mandibular dental midline was deviated to the right by 1 mm. Several caries were detected, especially on occlusal surfaces (Figure 1). Panoramic radiographs showed that the four third molars were embedded in the bone with a normal shape. We also noted the two residual roots with periapical lesions at the #46 site, and #47 was in good condition (Figure 2). No pathology was seen in the temporomandibular joint. Medical history was negative.

Treatment objectives

The primary goal of orthodontic treatment was to restore the occlusal function at the edentulous area. The second goal was to improve the patient's lateral facial profile and midline deviation.

Treatment alternatives

The following treatment options were considered. (1) Comprehensive orthodontic treatment and molar protraction, (2) comprehensive orthodontic treatment and implant restoration of #46 in adulthood, and (3) space closure with minor tooth movement (protraction of #47 and #48). Dental implant restorative treatment for tooth #46 was not considered at that time due to the patient's adolescent age. The first two alternatives can improve the convex facial profile and restore the occlusal function at the edentulous region. We discussed these treatment options with the patient, and she preferred the third option due to the shorter treatment duration. Informed consent was obtained from the patient for publishing the case details and images.

Treatment progress

The treatment was carried out in three stages: (1) extraction, osteotomy of cancellous bone, and insertion of mini-implant; (2) protraction of #47 through Albert loop appliance; and (3) alignment of #48 through segmental archwires.

**Figure 3:**
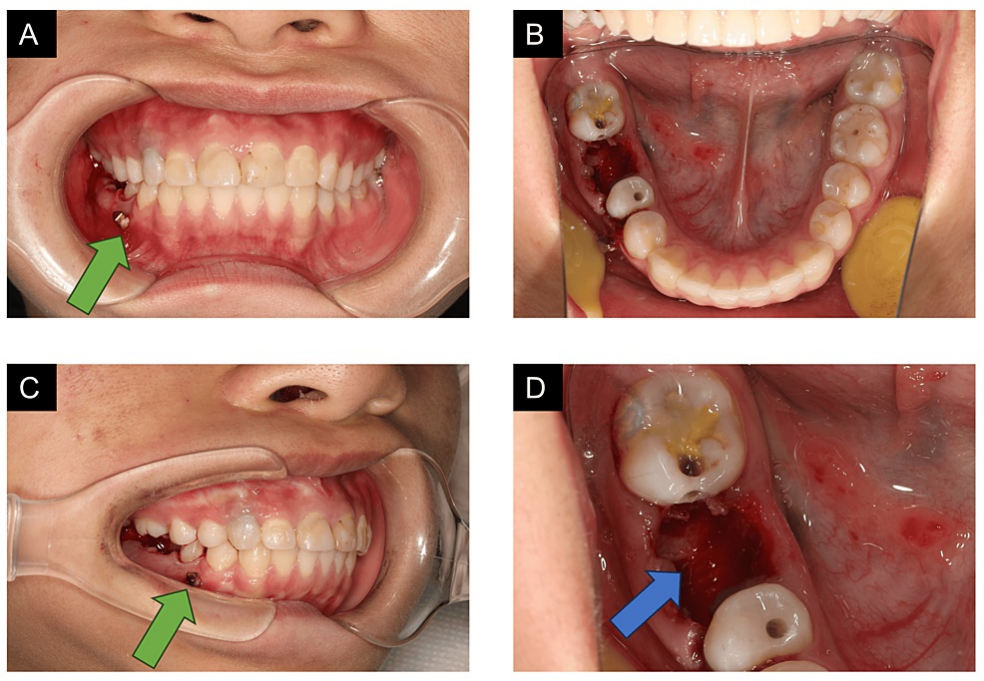
Extraction of tooth #46, piezosurgery, and insertion of the mini-implant. (A) The mini-implant was inserted and angulated to the alveolar bone. (B) Extraction of tooth #46. (C) The mini-implant was placed between the roots of #44 and #45. (D) After piezosurgery, the alveolar crest of root bifurcation was removed. The green arrow pointed to the mini-implant; the blue arrow pointed to the piezosurgery place.

According to the treatment plan, caries involving all teeth were treated before orthodontic treatment. After the residual roots of #46 were extracted, the cancellous bone at this region was removed through piezosurgery (Figure 3). A mini-implant (diameter, 1.4 mm; length, 8 mm; VectorTAS, Ormco) was inserted between the first and second premolars. An Albert loop appliance was fabricated and mounted for the protraction of the mandibular right second molar. A radiographic examination was then performed to rule out root contact by the mini-implant.

The Albert protraction loop appliance was made of 0.019 × 0.025-inch stainless steel wire. It consisted of a fixation hook mesially, a traction hook, one or two running loops, and a long cantilever arm posteriorly.

**Figure 4:**
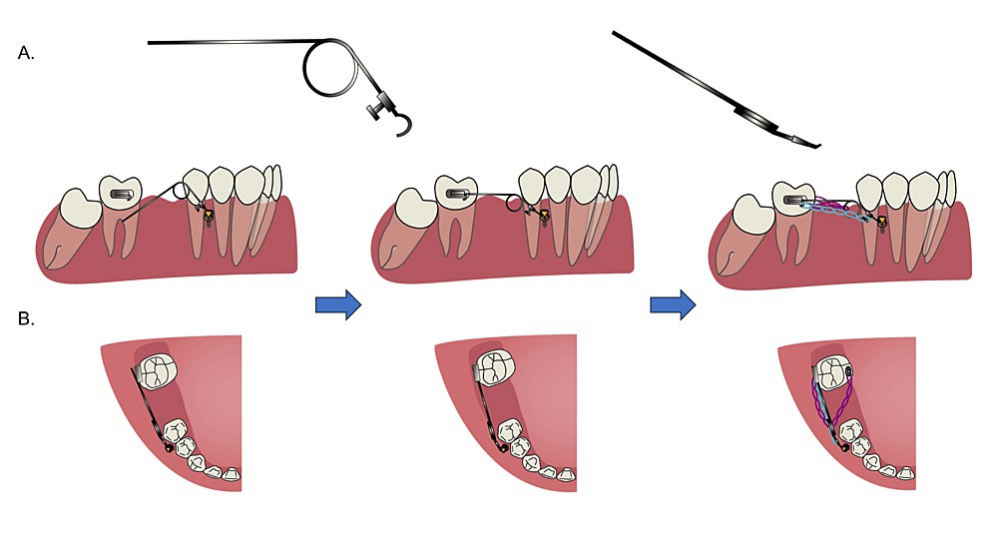
Albert protraction loop appliance. (A) Albert protraction loop from the front and top views. (B) Activation of the loop and application of elastomeric chains. Image credit: Xuechun Yuan.

When inactivated, the appliance forms an acute angle. To activate the Albert protraction loop appliance, the cantilever arm should be inserted into the buccal tube and the fixation hook should be cemented on the mini-implant. Also, it was significantly important that the cantilever arm should be parallel to the occlusal plane. Then two elastomeric chains were also applied (Figure 4).

**Figure 5:**
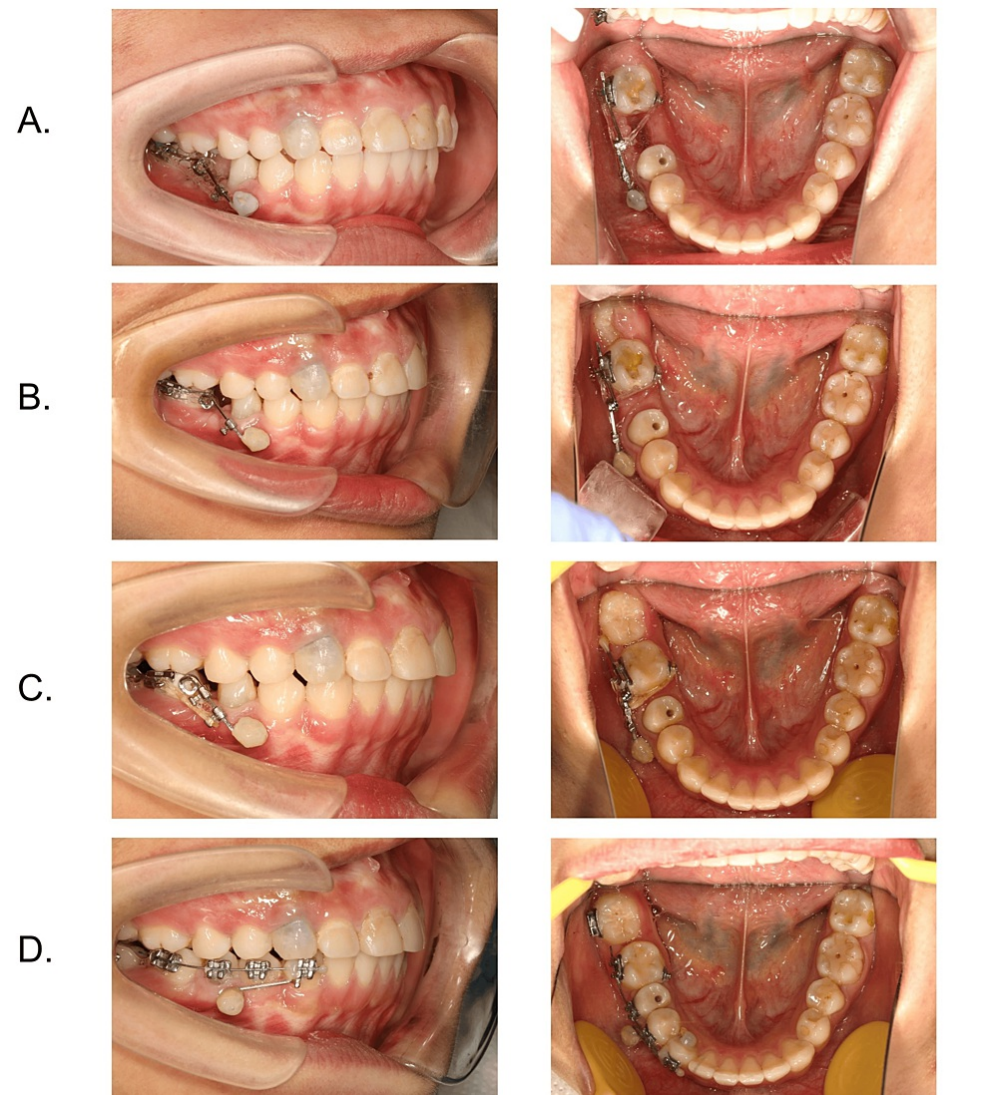
Protraction process. (A-C) Bodily mesial movement of #47 was achieved and #48 erupted spontaneously. (D) Alignment of #48 through brackets and archwires.

We replaced two elastomeric chains at each follow-up to make sure there was a continuous force to protract #47. Tooth #47 was protracted in less than 14 months. Within the procedure, #48 erupted spontaneously and moved and drifted mesially (Figure 5).

After 14 months, the Albert loop was removed and brackets and segmented archwires were placed to align tooth #48 and adjust the occlusion. Once #48 was aligned and leveled, the brackets, archwire, and mini-implant were removed. The patient then underwent restorative treatment for tooth #45 (Figure 5).

Treatment results

**Figure 6:**
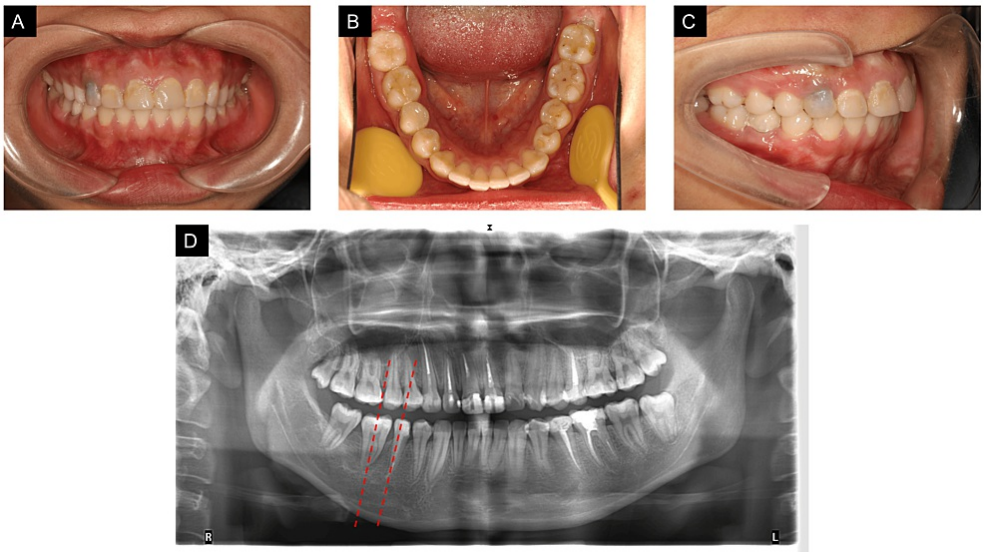
Posttreatment intraoral photographs and posttreatment panoramic radiographs. (A-C) Intraoral photographs. (D) Panoramic radiograph. It was taken before the #45 restoration. The angle between the two red lines was 1.5°.

The patient's overall treatment time was 17 months, including protraction of #47 (14 months), alignment of #48, and occlusal adjustment (three months). Tooth #46 was restored with significant improvement in occlusion. Based on the post-treatment intraoral evaluation, it was observed that #46 was restored perfectly. We also achieved a Class I molar relationship on the right side. Panoramic radiograph showed acceptable root parallelism of tooth #47: the angle between #47 and #45 was 1.5° (less than 7°), indicating good root parallelism. Also, there was no remarkable root resorption of #47, and it was protracted mesially for 10.43 mm (Figure 6).

## Discussion

Protraction of mandibular molars is a challenging task that necessitates adequate anchorage and meticulous biomechanical design. Owing to the large root surface of the mandibular molar and the high bone density of the mandible, it is difficult to protract mandibular molars without anchorage reinforcement. Mandibular molar protraction through conventional biomechanics often leads to the loss of anterior anchorage, resulting in reciprocal retraction of incisors during molar protraction [10]. Mini-implants have demonstrated their utility in reinforcing the anterior anchorage or serving as the direct anchorage for molar protraction. Notwithstanding their advantages, certain limitations persist. These limitations stem from achieving simultaneous uprighting and protraction forces with a singular force application, potentially resulting in lingual tipping and rotational movements of the molars [10,11]. Therefore, researchers have used U-curve, upright spring, upright jet, and traction from removable appliances to prevent lingual tipping [3-5,8]. These devices were mainly applied with comprehensive orthodontic treatment and mostly solved the issue by adding an uprighting force. However, the rotation of the molar is more easily neglected: the protraction will have a mesial rotation on the molar. Those side effects caused roundtripping, resulting in prolonged treatment duration.

**Figure 7:**
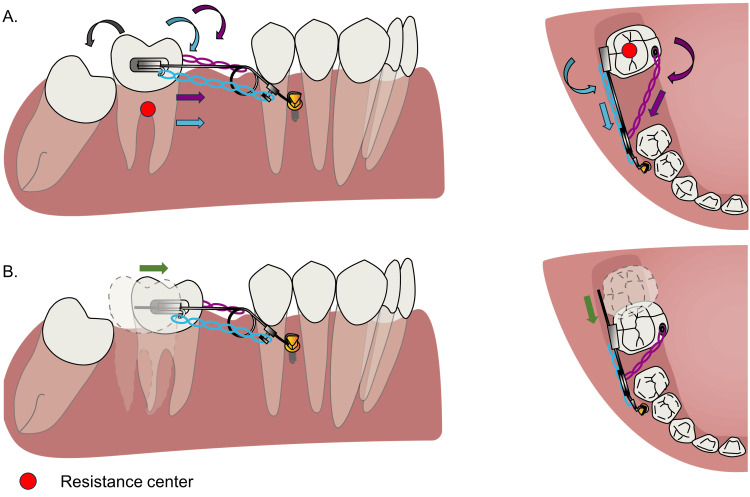
Biomechanics of the protraction system. (A) On the buccal side, elastomeric chains provided mesial forces and clockwise moments, while the activation of the Albert protraction loop provided a counterclockwise moment. On the occlusal view, the elastomeric chain on the buccal side provided a mesial force and counterclockwise moment, while the elastomeric chain on the lingual side provided a clockwise moment to counteract with the moment generated from the buccal side. (B) This results in the bodily movement of #47. Image credit: Xuechun Yuan.

In this case, a mandibular molar protraction device, the Albert loop was proposed to solve the aforementioned problem. The biomechanics of this device can be divided into the following three parts. From the sagittal view, the force from the elastomeric chains on the buccal and lingual sides will both provide a protraction force and a clockwise moment, and the latter will lead to the mesial inclination of #47. When the Albert protraction loop was activated, the cantilever arm provided a counterclockwise moment, which can counteract that clockwise moment. From the occlusal view, the elastomeric chains on the buccal side had a counterclockwise moment on the tooth, whereas the one from the lingual side provided a clockwise moment, which can counteract the counterclockwise moment from the buccal side. Furthermore, the cantilever arm was also acting as the guiding arm. The arm was parallel to the occlusal plane and the dental arch, facilitating the molar to move along the arm. In general, bodily movement of #47 can be achieved. To be noted, the Albert protraction loop needs to be pressed downward until the cantilever arm is parallel to the occlusal plane; otherwise, the elevated cantilever arm may exert an upward force on the molar, potentially leading to extrusion (Figure 7).

**Figure 8:**
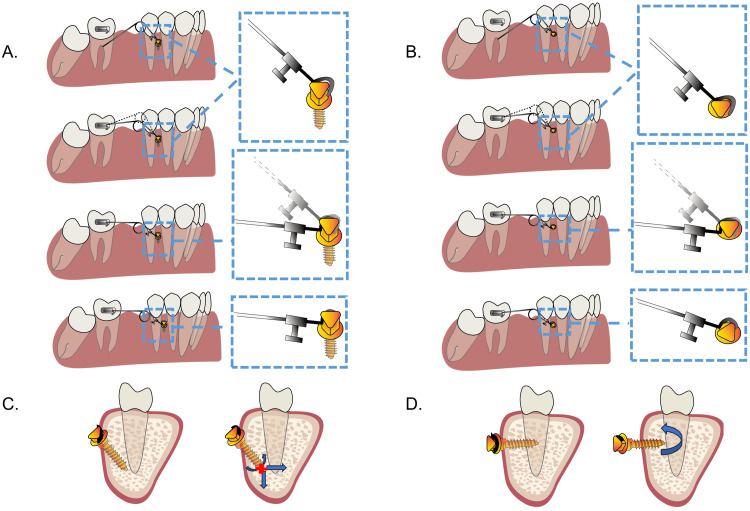
Angulated insertion of the mini-implant vs. straight insertion of the mini-implant. (A and C) Angulated insertion. The derotation force generated by the protraction device can be decomposed.  (B and D) Perpendicular insertion. It is easier to derotate the mini-implant. Image credit: Xuechun Yuan.

It is worth emphasizing the importance of the angulated insertion of the mini-implant. To activate the Albert protraction loop, the cantilever arm needs to be pressed apically. It generates a rotational force on the mini-implant, which is a clockwise moment. This derotation force on the mini-implant may be disastrous because the mini-implant is not resistant to rotational moment. To stabilize the mini-implant, it should be inserted toward the root at an angle of around 60° with the occlusal plane. By implementing this approach, the derotation force was alleviated, and we were able to gain more interradicular space and more cortical bone contact area for the mini-implant. In this way, the stability of the mini-implant was improved and the risk of adjacent teeth damage was mitigated (Figure 8).

Although some studies showed that an increase in the second molar protraction rate results in mesial tipping of the third molars, we achieved both bodily movement and high protraction rates at the same time. The Albert loop ensures the bodily movement of the molar by controlling the molar in all three directions, thereby avoiding mesial tipping and lingual rotation. Moreover, it prevents potential extrusion of the molar. Also, the bodily movement of #47 avoided any possible roundtripping. The utilization of the device, in combination with osteotomy, demonstrated remarkable efficiency in molar movement, achieving a displacement of over 10 mm within a span of merely 14 months in this particular case.

After extraction of #46, we performed osteotomy to reduce the bone resistance to accelerate the protraction. This was based on the Regional Acceleratory Phenomenon (RAP) reported by Frost. The effect on the bone is a reduction in regional bone density due to an increased remodeling space [12]. Based on RAP, various orthodontic modalities have been developed to accelerate tooth movement including periodontally accelerated osteogenic orthodontics, corticotomy, piezocision, etc. [12]. In addition to osteotomy, in recent years, there have been some researchers who have tried the acceleration of protraction of molars by surgical procedures. Han et al. performed a mandible corticotomy in dogs, leading to reduced treatment time and almost bodily movement of the posterior teeth [13]. Human trials by Kook et al. also proved the effectiveness of corticotomy. However, corticotomy involves flapping and may be uncomfortable [14]. Therefore, Al-Areqi et al. performed piezocision, that is, laser-assisted flapless corticotomy on both sides of the gap [15]. Piezocision was more effective compared to the control group: second molar movement distance doubled in the first two months after the operation. But the overall duration of protraction was reduced by only one month. Three-dimensional finite-element studies have demonstrated the beneficial effects of corticotomy and osteotomy in facilitating molar uprighting [16]. Of course, there are many objections raised regarding this approach [17]. Whether or not a physician uses surgically accelerated orthodontic treatment can be evaluated comprehensively to assess the patient's situation and needs. In this case, the patient underwent post-extraction osteotomy to remove the alveolar bone under the root bifurcation, which led to uniform bone density throughout the space, and accelerated tooth movement with the assistance of RAP.

 During the protraction of #47, #48 erupted and tipped mesially, possibly due to its potential to erupt (open apices before the treatment) and an increase in available posterior space. Spontaneous eruption of most third molars can occur as the mandibular second molar is moved mesially, but a small portion may not erupt. If the tooth is of horizontal angle or inverted impaction, the tooth would not erupt [18]. In addition to the angulation of the third molar, age and the Nolla stage of the third molar are influencing factors for its spontaneous eruption. It has been proved that the younger the age, the larger the third molar Nolla stage, and the closer the mandibular third molar is to the occlusal plane, the more it tends to move proximally to the neighboring teeth [19]. Studies also indicated that patients with impacted and mesially angulated third molars at pretreatment may have less alveolar bone resorption distal to the second molars following protraction [20]. The patient, in this case, had a medium horizontal angle of the #48 initial state, a high Nolla stage, a young age, and the presence of large eruption dynamics, so it had more chances to erupt spontaneously and move mesially following the protraction of the second molars. It has also been shown that the longer the period of traction, the more proximal-medial movement [18]. Practitioners should pre-evaluate the mandibular third molar before or during the protraction of the mandibular second molar, deciding whether or not to assist the eruption. 

## Conclusions

Successful molar protraction needs a carefully designed force-and-moment system with a good understanding of its biomechanics. The combination of the Albert protraction loop and mini-implant allows for more efficient protraction of the mandibular molars, avoiding mesial tipping and lingual rotation of the molars.
